# First evidence of wasp brood development inside active nests of a termite with the description of a previously unknown potter wasp species

**DOI:** 10.1002/ece3.6872

**Published:** 2020-10-06

**Authors:** Helder Hugo, Marcel G. Hermes, Bolívar R. Garcete‐Barrett, Iain D. Couzin

**Affiliations:** ^1^ Centre for the Advanced Study of Collective Behaviour University of Konstanz Konstanz Germany; ^2^ Department of Collective Behaviour Max Planck Institute of Animal Behavior Konstanz Germany; ^3^ Department of Biology University of Konstanz Konstanz Germany; ^4^ Department of Biology Universidade Federal de Lavras Lavras Brazil; ^5^ Museo Nacional de Historia Natural del Paraguay San Lorenzo Paraguay; ^6^ Department of Biology Universidad Nacional de Asunción San Lorenzo Paraguay

**Keywords:** brood development, *Constrictotermes cyphergaster*, Montezumia, Nasutitermitinae, symbioses, Vespidae

## Abstract

Potter wasps (Vespidae: Eumeninae) are known to exhibit not only sophisticated preying strategies but also a remarkable ability to manipulate clay during nest building. Due to a mixture of plasticity in building behavior and flexibility in substrate preferences during nest building, the group has been reported nesting in a variety of places, including decaying nests abandoned by termite species. Yet, evidence of wasps nesting inside senescent termite mounds is poorly reported, and to date, accounts confirming their presence inside active colonies of termites are absent. Here, we address a novel intriguing association between two species from the Brazilian Cerrado: a previously unknown potter wasp (nest invader) and a termite species (nest builder). Besides scientifically describing *Montezumia termitophila* sp. nov. (Vespidae: Eumeninae), named after its association with the termite *Constrictotermes cyphergaster* (Silvestri, 1901) (Termitidae: Nasutitermitinae), we provide preliminary information about the new species' bionomics by including (a) a hypothetical life cycle based on the evidence we collected and (b) a footage showing the first interaction between a recently ecloded wasp and a group of termites. In doing so, we attempt to provoke relevant discussions in the field and, perhaps, motivate further studies with the group. Finally, we describe a solution to efficiently detect and sample termitophilous species from termite nests, an intrinsic yet challenging task of any studies dealing with such a cryptic biological system.

## Graphical abstract

We describe a previously unknown potter wasp species, *Montezumia termitophila* sp. nov. (Vespidae: Eumeninae). Also, we provide the first evidence of wasp brood development inside active nests of the termite Constrictotermes cyphergaster (Silvestri, 1901) (Termitidae: Nasutitermitinae).

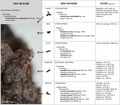

## 1 INTRODUCTION

A species’ ability to persist locally and over time depends on its capacity to successfully breed and give rise to subsequent generations. This definition seems to hold irrespective of major transitions in evolution (Maynard Smith & Szathmáry, 1995), being observed in solitary, gregarious, semisocial, and eusocial organisms. Among the variety of evolved strategies for developing immatures into adult individuals, nest building has evolved independently across taxa into many forms. The variation in nest architecture includes, but is not limited to, structures built by single individuals on top of pre‐existing nests (such as in the species here reported), conglomerates of reproductive cells assembled by semisocial organisms (Bohart & Stange, 1965; Lopes & Noll, 2019; West‐Eberhard, 2005), and large‐scale nests collectively built by massive eusocial colonies (Eggleton, 2010; Hölldobler & Wilson, 1990; Seeley & Morse, 1976). Yet, despite the variety of strategies, groups taxonomically independent and, therefore, evolving under seemingly unique selective pressures may still coincide in their nesting location. In fact, while a number of species settle in proximity and coexist harmoniously, in some cases in complete absence of interdependency (e.g., plesiobiosis, Kanizsai et al., 2013), a relatively smaller group is known to “cheat” by deliberately using a physical structure built by another species (Nash & Boomsma, 2008; Uboni et al., 2012). In termites, arguably one of the most successful builders in nature (Wilson, 1971), this pattern has been long and recurrently observed (Collins, 1980; Kistner, 1969, 1979, 1990; Mathews, 1977; Redford, 1984); nests built by a single builder species, and thus meant to house primarily nestmates, becoming a hub of opportunistic invasive species.

Because nest building is presumably a costly and time‐consuming process (Korb & Linsenmair, 1999), one would expect that species other than the builder would attempt to avoid such a commitment and take advantage of pre‐existing constructions, either “gently” (e.g., nest sharing) or “by force” (e.g., nest usurpation). Theoretically, while in the former approach invaders must find a way to stably coexist with their hosts, the latter only requires a hostile takeover in which the builder is killed off and replaced by the invader. Although practical, such a binary simplification should be considered carefully; in reality, the outcome of an association between species over time is more likely to transit in a continuum between the extremes. In either case, what seems fairly consistent is the fact that nest invaders are somehow lured by specific benefits associated with the host nest. Indeed, besides providing shelter, nests may be a pool of valuable resources that drive trophic interactions, particularly those exhibiting predatory (Jaffe et al., 1995) or parasitic habits (Yashiro & Matsuura, 2007).

In termites, builder species invariably exhibit mechanisms to prevent nest invasion, such as nestmate recognition (Clement & Bagneres, 2019) and a caste system in which colony members—namely soldiers—play a defensive role (Prestwich, 1984). At the same time, however, nest invaders have developed sophisticated sensory deception mechanisms to go unnoticed by their hosts (Nash & Boomsma, 2008), such as chemical and morphological mimicry. The existence of such mimicry strategies illustrate how evolution may shape nest invaders into true specialists, experts in deceiving the termite builder (examples in Cunha et al., 2015; Oliveira et al., 2018; Pisno et al., 2019; Rosa et al., 2018). The arms race between builder and nest invader, driven by adaptations and counteradaptations from both sides (Dawkins & Krebs, 1979), is thought to produce cases in which the symbiosis is taken to another level, where one of the species—if not both—ends up relying exclusively on the other for survival. Such a dependency on a host is exhibited by some secondary termite species (e.g., inquilines) and a number of other insects (e.g., termitophiles), which gain entry to the termite nest when host colonies are still alive. However, because a termite construction is likely to persist in the wild long after its colony dies, another great number of species opportunistically use abandoned nests as shelter (Costa et al., 2009), becoming occasional occupants (Wilson, 1971). Together, these facts seem to indicate that regardless of the current status of its colony, a single termite nest will most likely attract a large number of foreign species throughout its time of existence.

Potter wasps (Vespidae: Eumeninae), known for their striking ability to shape clay during nest building, have been reported nesting in a variety of substrates (Cowan, 1991; Garcete‐Barrett & Hermes, 2013; Hermes et al., 2015), including decaying nests of termites abandoned by the builder species (Batra, 1979). Curiously, to date, there is no evidence of nesting by wasp species inside active colonies of termites. Such a pattern, upon first glance, could be interpreted as a result of nesting preferences exhibited by the group. However, except for some knowledge of bees as “guests” in termite nests (for examples of species in Apidae, see Carrijo et al., 2012), little is known about the remainder clades of Hymenoptera, such as Vespidae, which may ultimately contribute to a misrepresentation of the habits exhibited by the group.

In this study, we describe a new species of potter wasp from the Brazilian Cerrado, *Montezumia termitophila* sp. nov. (Vespidae: Eumeninae), and report the first record of brood development by a wasp species inside active nests of the termite *Constrictotermes cyphergaster* (Silvestri, 1901) (Termitidae: Nasutitermitinae). In addition to reporting the unusual nesting site of the new wasp species, we discuss relevant yet poorly understood proximate causes of such a development. Additionally, to add notes on the species’ biology and potentially stimulate further studies, we propose a life cycle for the new wasp based on the evidence we collected. Finally, we provide a protocol for detecting and sampling termitophilous species from termite nests, an intrinsic but challenging task that, if poorly conducted, may contribute to misrepresenting the diversity of several cryptic organisms, such as the new wasp we discovered.

## 2 MATERIALS AND METHODS

### 2.1 Terminology

Conventionally, animals invading nests of social organisms are referred by terms that provide a direct reference to the host group they are found with (e.g., “sphecophiles” with wasps, “melitophiles” with bees, “myrmecophiles” with ants, “termitophiles” with termites; Wilson, 1971). Yet, in Termitology (i.e., study of termites) two terms have been adopted when referring to organisms in association with host termites: “termitophiles” and “termitariophiles” (Kistner, 1969, 1979, 1990). While the former indicates species with association with the host termite colony, the latter indicates species in association with the physical structure of termite nests (i.e., termitarium), not with colony individuals. Recently, this distinction has become clearer with studies in which the authors provide an explanation for choosing either one of the terms (Carrijo et al., 2012; Cunha et al., 2015; Pisno et al., 2019; Rosa et al., 2018). Here, we explicitly follow Kistner’s (1969) original concept of termitophilous associations which defines “termitophiles” as nontermite species that either (a) live inside galleries of host termite nest or (b) have obligatory association with the host termite colony. This mention is important to clarify which concept we considered when defining the specific epithet for the new species, choosing “termitophila” instead of “termitariophila.”

The terminology we considered for referring the new eumenine wasp also deserves comment. As clade of primarily solitary vespids, the eumenines have been traditionally treated as closely related to the social subfamilies within the Vespidae (Pickett & Carpenter, 2010). However, phylogenomic studies (Bank et al., 2017; Piekarski et al., 2018) have challenged the traditional view on the phylogenetic relationships among vespid subfamilies, with the eumenines comprising a paraphyletic assemblage regarding the Polistinae + Vespinae. This is paramount in understanding the evolution of life strategies and nesting behaviors, since the different phylogenetic scenarios may lead to diverging conclusions regarding the origin and maintenance/spread of different nesting strategies. Since we are considering the evolution of nesting behavior herein, we will use the classification of Hermes et al. (2014), which includes the zethines among the Eumeninae, in order to provide a broader array of nesting strategies displayed by this group of wasps.

### 2.2 Host termite species

*Constrictotermes cyphergaster* (Silvestri, 1901) is a Neotropical termite species that builds typical arboreal nests (Krishna et al., 2013; Vasconcellos et al., 2007) and is widespread throughout South America (Mathews, 1977). With nocturnal foraging habits and diet mostly based on lichens (Barbosa‐Silva et al., 2019) and tree bark (Moura et al., 2006), the species has drawn some attention due to its close association with nest invaders, such as *Inquilinitermes microcerus* (Silvestri, 1901) and *Inquilinitermes fur* (Silvestri, 1901) (for examples of studies, see Cristaldo et al., 2012, 2014, 2015, 2016; Cruz et al., 2018; Cunha et al., 2003; DeSouza et al., 2016; Florencio et al., 2013; Hugo et al., 2020; Rodrigues et al., 2018; Santos, 2016). Because of their limited constructive abilities (Emerson, 1938), these invader termite species are known to rely exclusively on nests of *C. cyphergaster* to establish their colonies, being not found anywhere else to date and, therefore, classified as “obligatory inquilines” (Mathews, 1977).

Mechanisms underlying obligatory termite–termite associations are supposedly specific to each host–inquiline pair. For *I. microcerus*, in particular, colonies are typically found cohabiting nests of *C. cyphergaster* above a certain nest size, supposedly 13 L in termitarium volume according to Cristaldo et al. (2012). The species is also characterized by a reduced number of termite soldiers (Cunha et al., 2003) and an uncommon lack of aggressiveness toward its host termites. In fact, this inquiline species has been reported to adopt evasive behaviors when receiving attacks and make use of fecal pellets to disrupt the host's aggression (Hugo et al., 2020). This nonthreatening behavior contrasts with the typical aggression seen among termite species (Shelton & Grace, 1996). Although known for a number of organisms, including colonial (Lhomme et al., 2012; Nehring et al., 2015; Pierce et al., 2002), gregarious (Aureli et al., 2002), and semisocial species (Baan et al., 2014; Gobush & Wasser, 2009; Thierry et al., 2008), submissive behavior still remains relatively uncommon among termites, given that most species tend to immediately reciprocate any sort of aggression received (Noirot, 1970; Prestwich, 1984; Shellman‐Reeve, 1997). Nests of *C. cyphergaster* are known to house, in addition to inquiline termites, termitophilous species such as *Corotoca melantho* (Coleoptera: Staphylinidae), a viviparous rove beetle that shares morphometric (Cunha et al., 2015) and chemical (Rosa et al., 2018) similarity with their host termites. These soft‐bodied, physogastric beetles exhibit reduced body proportions (Cunha et al., 2015) and cryptic habits (Pisno et al., 2019), which often turn detection and behavioral observation into challenging tasks. Yet, with adequate methods, relevant biological information about the group has been revealed, such as mechanisms underlying the nest invasion (Oliveira et al., 2018).

**Figure 1:**
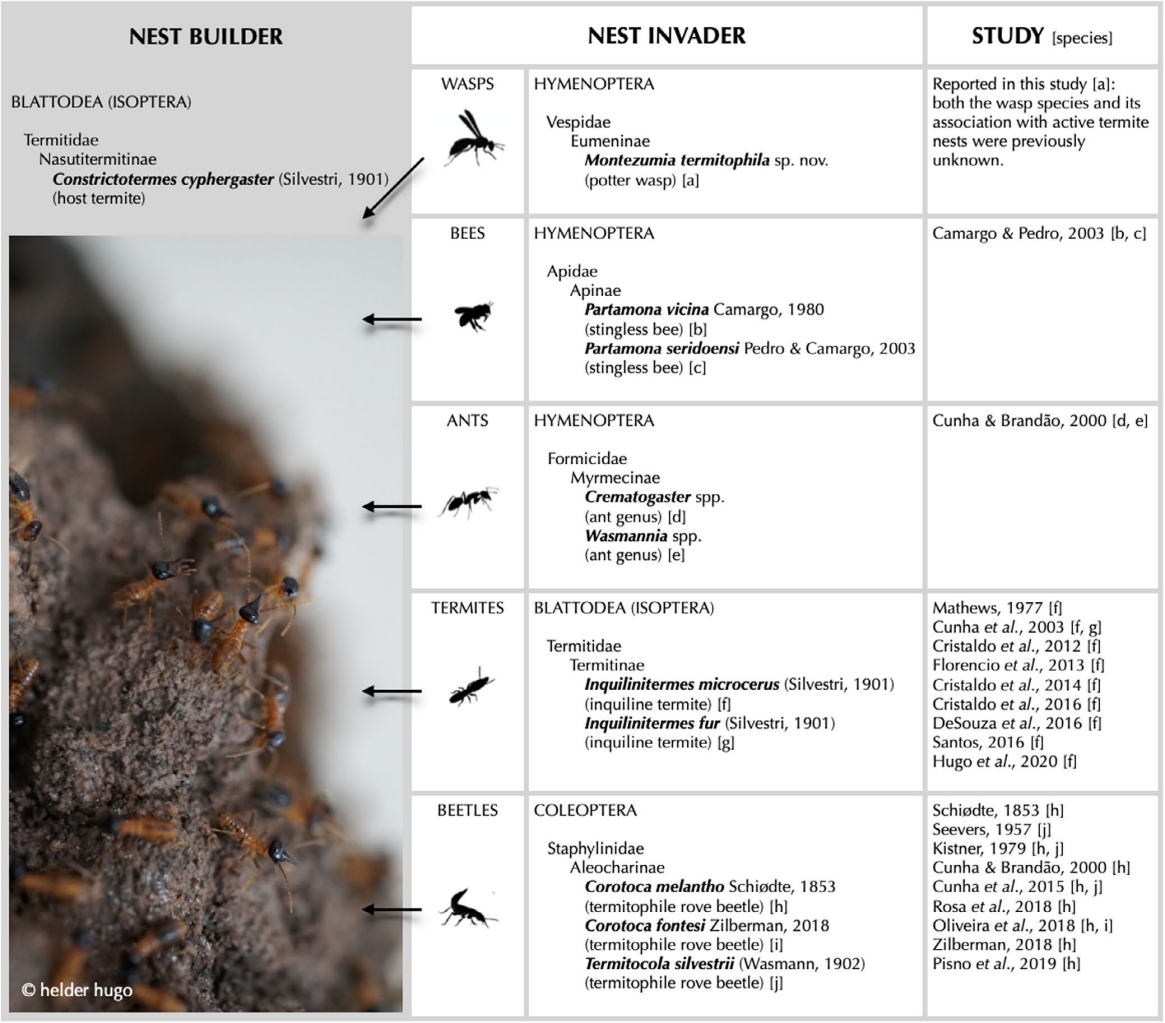
Organisms currently known to invade nests of the termite *C. cyphergaster*. Nest invaders are represented along with a brief taxonomical classification (from order to genus or species). The references for investigations also contain indications for the species reported (in squared bracket).

Recently, the number of studies investigating species in association with *C. cyphergaster* has increased. Figure 1 presents an overview of the organisms currently known to invade active nests of *C. cyphergaster*, with their taxonomical information. As shown, a handful of associations have been investigated, being inquiline termites (Blattodea: Termitidae) and termitophilous rove beetles (Coleoptera: Staphylinidae), the groups receiving most attention. In a comprehensive review of groups belonging to Hymenoptera: Apidae (e.g., Apinae, Centridini, Euphorini, Eucerini, Colletinae, Megachilinae), Carrijo et al. (2012) have compiled a number of bee species occurring inside termite nests. In contrast, there is no record to date of wasps being found inside active termite nests.

### 2.3 Potter wasps

The nesting behaviors exhibited by potter wasps have been traditionally classified into three types: (i) excavators, where females dig directly into dry substrate and moisten it with regurgitated liquid during excavation; (ii) renters, where females use pre‐existing cavities; and (iii) builders, where females collect dry earth and mix it up with regurgitated liquid to make mud cells (Cowan, 1991; Iwata, 1976; Maindron, 1882). However, potter wasp species present plasticity regarding these nesting strategies (Cooper, 1979; Krombein, 1979), and nest architecture may vary as well (e.g., Hermes et al., 2015). Additionally, some species make use of vegetal matter during nest building, either incorporating leaf particles to provide camouflage to nest (Hermes et al., 2013) or using plant material in cell construction itself as presented by some zethines (Bohart & Stange, 1965; Claude‐Joseph, 1930; van der Vecht, 1981; van der Vecht & Fischer, 1972). Females of eumenine wasps usually nest solitarily, but there are accounts reporting the aggregate behavior of nesting collectively, where several females share one nesting site, but each female tends their own brood (Bohart & Stange, 1965; Lopes & Noll, 2019; West‐Eberhard, 2005). In fact, some of these accounts are of particular interest, because they are exhibited by species in the genus *Montezumia* de Saussure. West‐Eberhard (2005) observed the nesting behavior of *M. cortesioides* Willink, 1982, in which several females shared a mud nest with neither division of labor nor hierarchical castes. However, some behaviors may precede the transition from solitary to social life, such as female competition for empty cells, progressive provisioning, and some females remaining in the nest for longer periods than others (allowing prey theft). Similar accounts were provided by Lopes and Noll (2019), with less detail, for the species *M. brethesi* Bertoni, 1918.

Associations between potter wasps and termite species have been mentioned a few times in the literature, yet vaguely supported by evidence. Batra (1979) has previously found in India the wasp species *Anterhynchium abdominale* subspecies *bengalense* (de Saussure, 1855) (Vespidae: Eumeninae) in a deteriorating mound of the termite *Odontotermes obesus* (Rambur, 1842). In a short communication, this author describes the positioning and morphology of wasp brood cells along the walls of exposed ventilation shafts, in a severely eroded and queen‐less mound. Reports of senescent termite nests without active colonies providing shelter to other species are various (e.g., Collins, 1980; Costa et al., 2009; Redford, 1984). Yet, to date, our study is the first to provide evidence of brood development by a wasp species inside active colonies of a termite species (*C. cyphergaster*).

### 2.4 Study site, sampling, and nest inspection

We collected 13 *C. cyphergaster* nests from one site in the Brazilian Cerrado (Ratter et al., 1997) near the municipality of Divinópolis (20°08ʹ20″S, 44°53ʹ02″W), State of Minas Gerais. This area is classified as ‘equatorial savanna with dry winters’ (Kottek, Grieser, Beck, Rudolf, & Rubel, 2006). The 13 termite nests, distributed sparsely in an area of 20 hectares and never neighboring one another, were collected in March 2018 (between 08:00 and 12:00) and immediately transported to the headquarters of property. Then, they were kept in individual cages for 5 days until nest inspection (Figure S2: 5). These cages, made of cubic wire frames surrounded by a thin tulle fabric, were designed to contain adult wasps after eclosion. As for verifying whether *M. termitophila's* nesting takes place entirely inside *C. cyphergaster* nests, it would be necessary to (a) obtain empirical evidence of adult wasps ecloding from the inside of termite nests and (b) document the presence of wasp's offspring in termite nests, by detecting wasp brood cells integrated between termite galleries containing wasp immature stages. Thus, to search for immature stages of wasps (eggs, larvae, and pupae) we thoroughly inspected the content of termite nests by peeling off its surface in order to expose subsequent layers of nest galleries (Figure S2: 5). To prevent damaging the biological material, we used scoopulas (stainless‐steel spatulas of application in chemistry) and collected individuals using entomological tweezers. As opposed to the method of merely breaking *C. cyphergaster* nests into smaller pieces and superficially checking for termitophiles (as conducted in previous studies with the species), the protocol mentioned above allows a more refined inspection with preservation of nest structures. Only by preserving such structures, we were able to later document and elucidate the positioning and architectural features of wasp brood chambers inside the termite nest.

### 2.5 Recording wasp–termite interaction

To add notes on the nature of the association between *M. termitophila* and *C. cyphergaster*, we observed the interaction between the wasp species and both termite workers and soldiers immediately after its eclosion, to identify possible interspecific aggression. To do so, we recorded a short video (six minutes) of the interaction between a female wasp, later defined as the holotype, and a group of 16 termites in 3:1 worker‐to‐soldier caste ratio (12 workers + 4 soldiers). The recording arena was set in a glass Petri dish of 60mm diameter lined with paper at the bottom and covered with a transparent lid, and the video was recorded in 4K (50 fps) under visible spectrum of light (with LEDs) using a Sony Alpha 7S camera equipped with a Zeiss Batis 25mm f/2 lens. The room temperature during species manipulation and video recording was set to 25 ± 1°C. To prevent excessive desiccation of termites, individuals were kept inside their nest until the beginning of recordings. We filmed only the first interaction between *M. termitophila* and *C. cyphergaster*. Because during manipulation three of the four adult wasps found managed to escape, we decided to preserve the remainder female (which was later defined as the holotype) for species identification and not conduct more trials. Although insufficient for statistical analysis, which is beyond the scope of this study, this behavioral record is meant to provide supplementary biological information. As such, it provides a first tentative empirical evidence for a mutual lack of aggression between a recently ecloded *M. termitophila* wasp and its host termites.

## 3 RESULTS

Our results constitute of two main parts: (i) a taxonomic account and (ii) a commentary on the new species’ natural history, both contextualized with the current knowledge on Eumeninae. In addition to formally describing *Montezumia termitophila* sp. nov. (Vespidae: Eumeninae), we present collected evidence that allows us to formulate a new hypothesis, that of adults in this wasp species explicitly use active nests of the termite *Constrictotermes cyphergaster* (Silvestri, 1901) (Termitidae: Nasutitermitinae) for brood development and, therefore, completion of its life cycle.

### 3.1 Taxonomic account

#### 3.1.1 *Montezumia termitophila* Hermes & Garcete‐Barrett, new species (Figure 2)

##### Diagnosis

**Figure 2:**
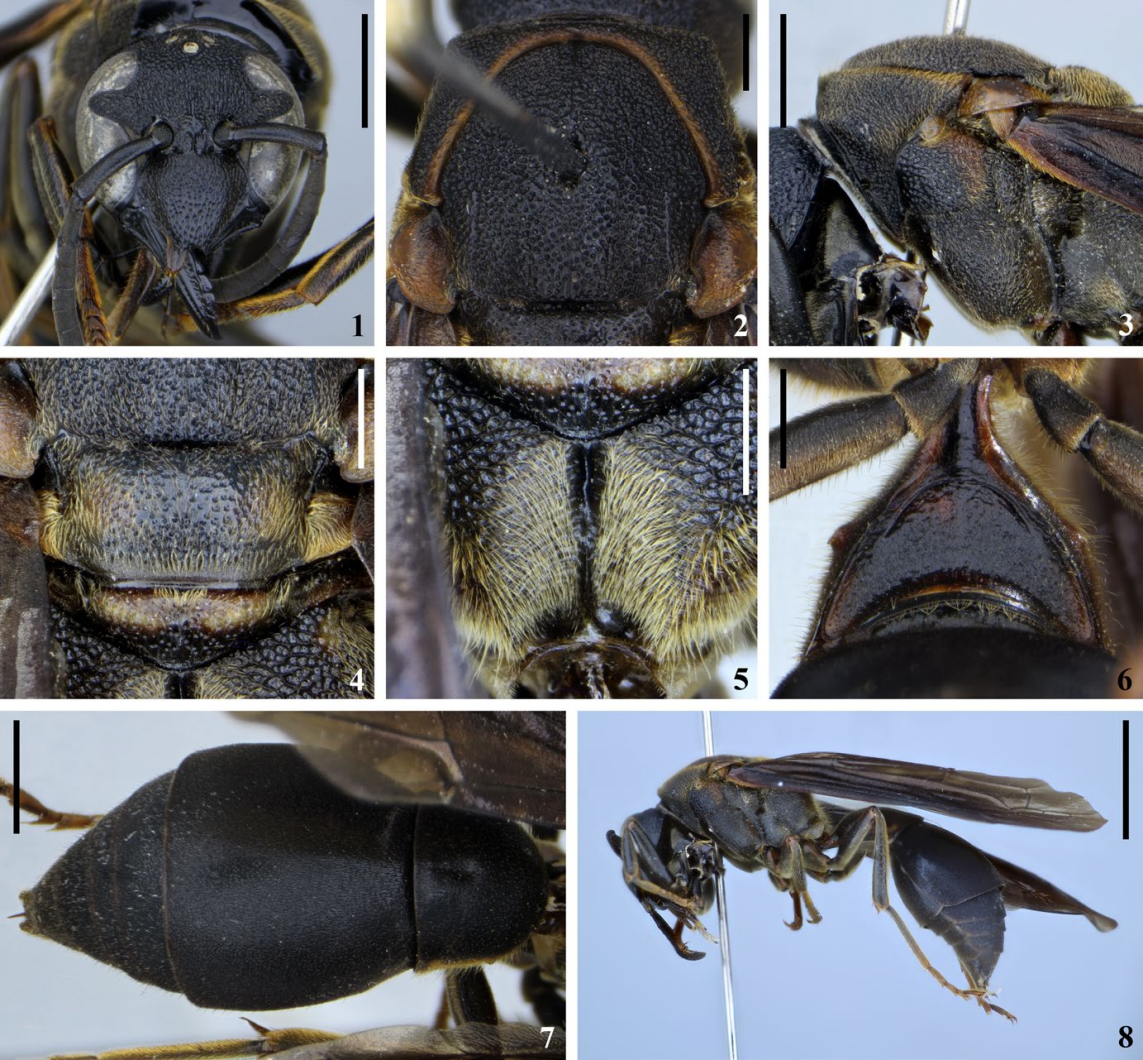
*Montezumia termitophila* new species, Holotype female. (1) Head in frontal view, scale = 2 mm; (2) pronotum and mesoscutum in dorsal view, scale = 1 mm; (3) pronotum and mesepisternum in lateral view, scale = 2 mm; (4) scutellum and metanotum in dorsal view, scale = 1 mm; (5) posterior surface of propodeum, scale = 1 mm; (6) metasomal sternum I, scale = 1 mm; (7) metasomal terga in dorsal view, scale = 2 mm; (8) habitus, scale = 5 mm

**Figure 3:**
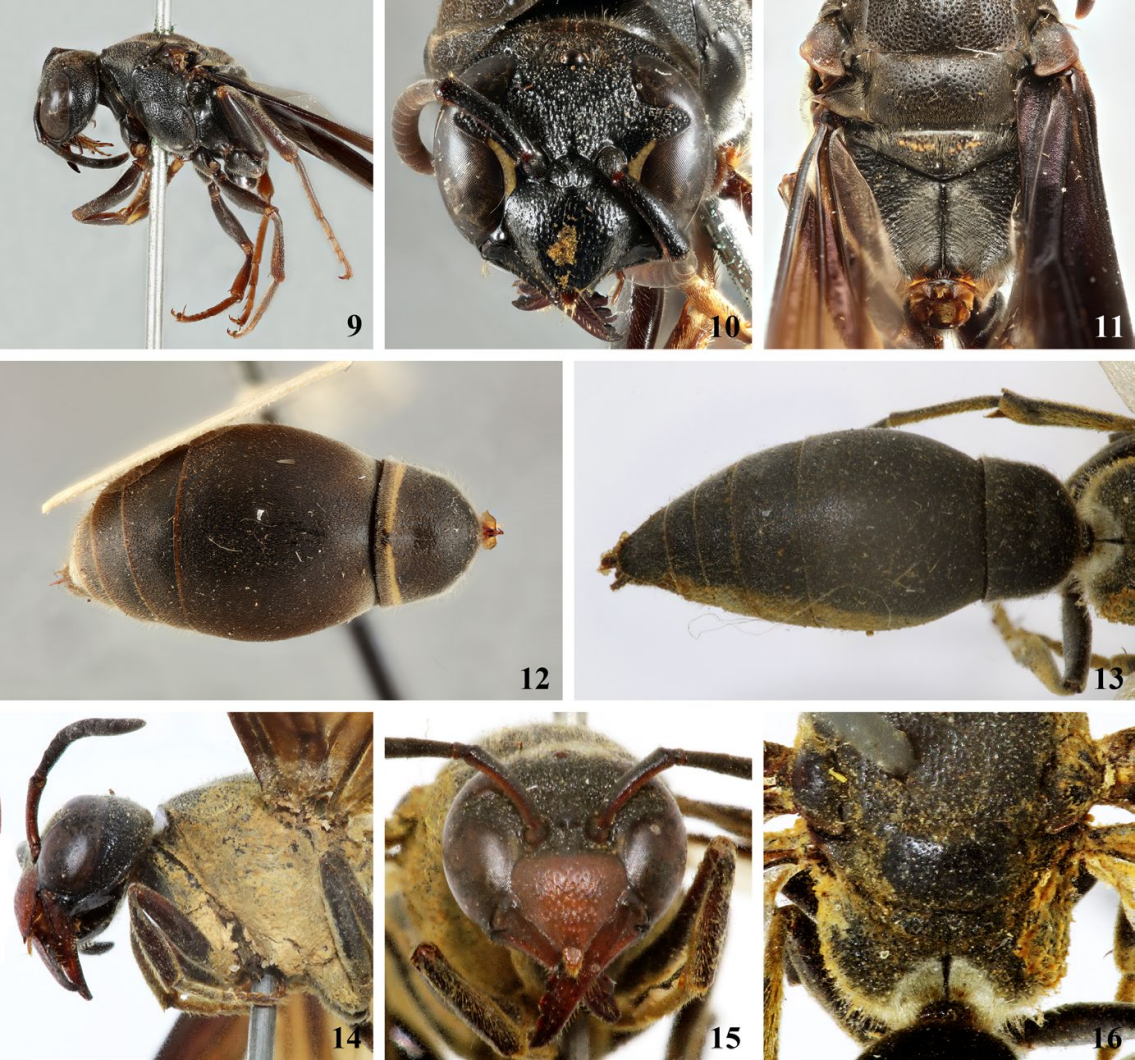
Two species closely related to *M. termitophila*. (9–12) *Montezumia grossa*, Paratype female. (9). Head and mesosoma in lateral view; (10) head in frontal view; (11) scutellum, metanotum, and posterior surface of propodeum in dorsal view; (12) metasomal terga in dorsal view. (13–16) *Montezumia morosa*, Lectotype female. (13) Metasomal terga in dorsal view; (14) head and mesosoma in lateral view; (15) head in frontal view; (16) scutellum, metanotum, and posterior surface of propodeum in dorsal view [Scale variable]

*Montezumia termitophila* **new species** belongs to the *M. infernalis* (Spinola, 1851) species group sensu Willink (1982). An emarginated clypeal apex (Figure 2:1), the lack of a keel in the mesepisternum right below the pronotal lobe (Figure 2:3), the lack of a humeral carina on the pronotum (Figure 2:3), and the metanotum with transverse toothed crests (Figure 2:4) are features that place the species within the mentioned group of species. Within the *M. infernalis* group, *M. termitophila* readily runs into couplet 48 of Willink's (1982) key. Furthermore, the species keyed to in the previous couplets all possess a smooth posterolateral margin in the propodeum when seen from above, which is somewhat angled in *M. termitophila*. Also, species such as *M. nigra* (Zavattari, 1912), *M. ignobiloides* Willink, 1982, *M. grossa* Willink, 1982 (Figure 3:9–12), *M. morosa* de Saussure, 1852 (Figure 3:13–16), *M. trinitata* Willink, 1982, and *M. obscura* Zavattari, 1912 are all part of a mimetic complex resembling the eusocial *Polybia ignobilis* (Haliday, 1836). The color pattern also differs between *M. termitophila* and *M. marthae* de Saussure, 1875, the latter presenting the metanotum, propodeum, and metasomal tergum I almost entirely yellow. *Montezumia termitophila*, in fact, resembles the eusocial *Agelaia angulata* (Fabricius, 1804) and the eumenine *Pachymenes ater* de Saussure, 1852 regarding body color, which is dark brown with yellow marks lacking almost entirely. Finally, *M. termitophila* differs particularly from *M. trinitata* by the lack of an apical lamella on metasomal tergum II (present in *M. trinitata*) and by the brownish body color with golden pilosity especially evident on scutellum, metanotum, and propodeum (black ground color and brownish pubescence only slightly evident in *M. trinitata*).

##### Description

**Figure 4:**
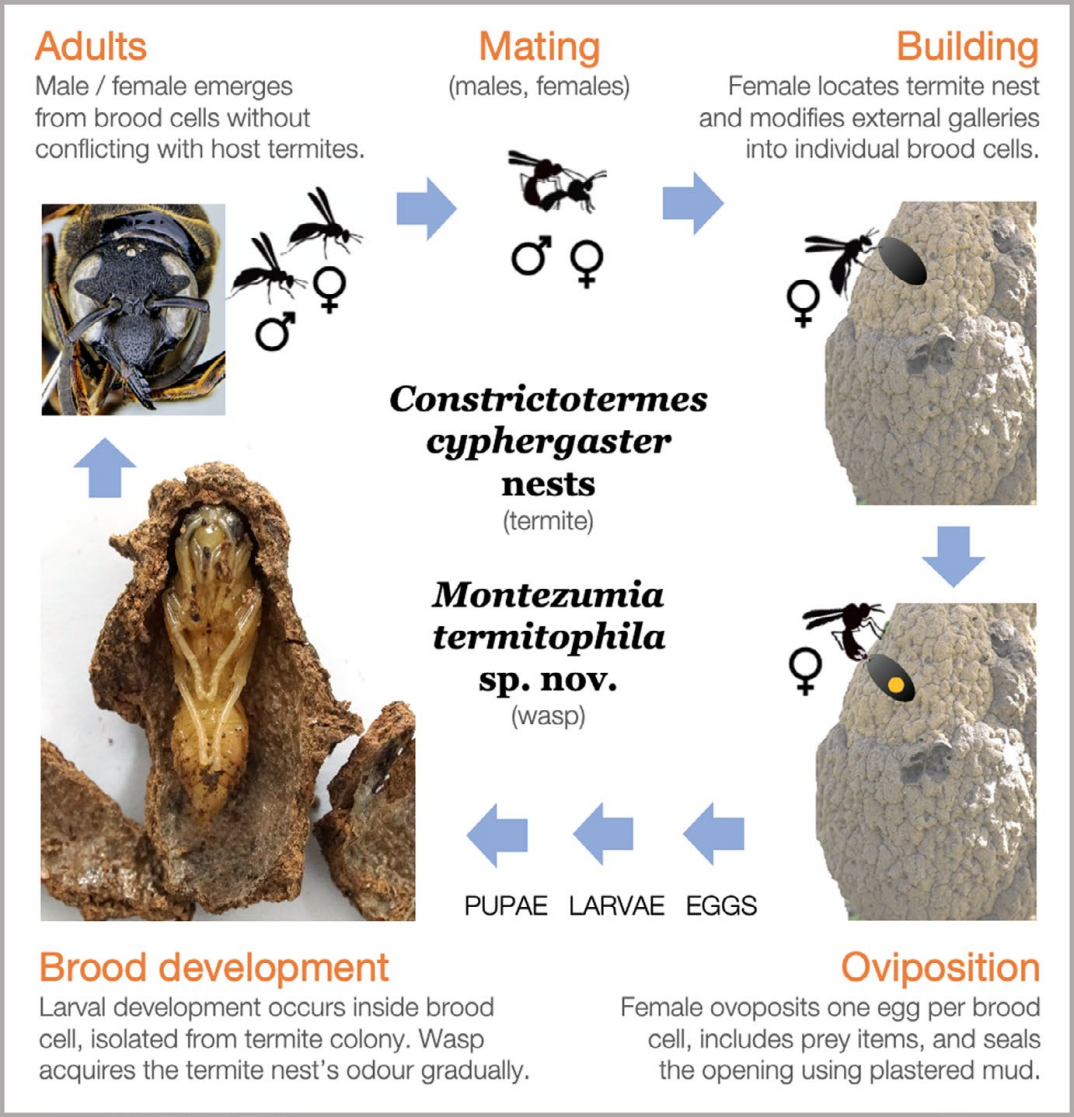
Life cycle proposed for *M. termitophila* Hermes & Garcete‐Barrett new species. Behaviors that were not explicitly observed in this study were based on the information available for other eumenines. Illustrative elements are simplified and not necessarily presented in proportional scale

Holotype female. **Color**: Body color dark‐brownish with light‐brownish markings (Figure 2:1–8) as follows: stripe on metanotum dorsally adjacent to mesoscutum; pronotal lobes entirely; marks on upper portion of mesepistermum; tegulae entirely; spots on lateral portions of scutellum; basal stripe on metanotum; lateral surface of mid and hind coxae; all tarsi entirely. Yellow thin stripes on anterior surface of fore tibiae. Brownish to ferruginous stripes on lateral portions of metasomal tergum I, becoming light yellowish toward the apex of the tergum. **Structure**: Body length from head to apex of T1 approximately 15 mm; forewing length 16 mm. Clypeus wider than long, apex emarginate with teeth evident; interantennal longitudinal carina present, with transversal arc‐like ramifications at mid antennal sockets; cephalic foveae slightly evident, surrounding area little differentiated; occipital carina well developed and uniform along entire length; pronotal foveae conspicuous; humeral region somewhat angled near pronotal carina, but not forming a ridge or lamella; pronotal carina well developed and uniform along entire length; mesepisternal carina present and reaching the dorsal sulcus; mesepisternum without keel right below the pronotal lobe; notaulices on mesoscutum slightly indicated, parapsidal lines present; sulcus between mesoscutum and scutellum ridged and deeper medially; metanotum with transverse toothed crests, posterolateral surfaces of propodeum somewhat angled dorsally; medium sulcus of posterior surface of propodeum deep on upper portion; median carina of posterior surface of propodeum present on lower portion; T1 longer than wide, with a shallow pre‐apical fossa; S1 somewhat triangular, twice as wide as long; T2 without apical lamella. **Sculpture**: Punctures dense throughout entire head and mesosoma, contiguous. Clypeus striatopunctate toward apex. Frons slightly striate between antennal sockets and mid ocellus. Posterior surface of propodeum weakly striate medially. Punctures smaller on metasoma, sparser on T1. **Pilosity**: Golden erect pilosity on most of head and mesosoma, especially dense on scutellum, metanotum, and propodeum. Golden erect pilosity on metasoma restricted to sterna and sides of T1; remainder of the metasoma with scattered short and very sparse pilosity. **Male**: Males were observed and photographed emerging from termite nests, but collection of voucher specimens for identification was unsuccessful (documentation provided in the Appendix). **Etymology**: The specific epithet is allusive to the fact that *M. termitophila* resides within nests of *C. cyphergaster* termites throughout its brood development, with no signs of conflict between both species. **Type Material**: Holotype female at *Coleção Entomológica da Universidade Federal de Lavras*, Minas Gerais, Brazil (CEUFLA). **Type locality**: Specimen collected on 25 March 2018, by one of the authors (HH) from a Brazilian Cerrado site near the municipality of Divinópolis (20°08ʹ20″S, 44°53ʹ02″W), State of Minas Gerais. The Holotype female was found originally in a brood cell inside an arboreal nest of the termite *C. cyphergaster* still in larval stage (pupa shown in Figure 4). The brood cell was carefully removed from the termite nest, conditioned in a separate container, and kept under controlled temperature and humidity until the wasp's eclosion. **Additional material examined**: Closely related species to *M. termitophila* were examined and compared to enhance the hypothesis of the latter as new to science. One paratype female of *M. grossa* and the lectotype female of *M. morosa* were examined. The first was collected in British Guiana in 1937 and identified by the late Abraham Willink and is currently housed at the Natural History Museum in London (NHM). The second specimen was collected in Rio de Janeiro (Brazil) and bears no indication of collecting date. It was also identified by Abraham Willink and is housed at the *Muséum National d’Histoire Nautelle* in Paris (MNHN). Also, one of the authors (MGH) has recently published a phylogenetic investigation on the genus *Montezumia* (Hermes & Carpenter, 2012), which encompassed the examination of several species within the taxon, therefore reassuring that *M. termitophila* is in fact a species previously unknown.

### 3.2 Biological traits of *M. termitophila*

#### 3.2.1 Morphology, positioning, and content of brood cells

The inspection of 13 colonies of *C. cyphergaster* yielded detection of 23 wasp brood cells (Table S1). Given that we identified no other wasp species in the material collected, and because all brood cells found had the same structure and proportions (Figure 4, Brood development), we registered all of them as belonging to *M. termitophila*. Of these, most were inactive and lacked immatures (*n* = 19), and only a few (*n* = 4) were active, containing either a larva (*n* = 1) or pupae (*n* = 3). During nest inspection, the three pupae developed into adults and emerged from their cell within two days of the detection date, but these escaped when being photographed for documentation (Figure S2, individuals in 1, 2, and 4). Of these, individual 4 was confirmed as a male for exhibiting a hook‐shaped last flagellomere in the antennae apex, individuals 1 and 3 were confirmed as females. The sex determination of individual 2 was not possible due to the poor resolution of image.

The brood cells found were positioned mostly on the nest's surface (Appendix, Figure S1), not deeper than two to three layers of nest gallery, counting from the most external layer. In one of the termite nests containing wasp's brood cell, vegetal matter was found attached nearby a cell opening (Appendix, Figure S2: nest in 5), but without behavioral observation we are not in the position to tell whether this was placed by *M. termitophila* or not. Regarding the internal morphology of *M. termitophila* brood cells, they were mostly cylindrical and incorporated in the substrate, tightly attached to gallery walls (see example in Figure 4; brood development). None of the brood cells was interconnected to termite galleries by openings. Yet, they were all located near sections densely populated by termites of the worker caste (albeit soldiers were also present). Inside the brood cells, we detected plastered mud (i.e., mixture of saliva and soil) over the internal walls.

As for that their content, none of the wasp brood cells, including active and inactive ones, were found to contain remnants of prey. Without nest‐building observation, it is impossible to associate *M. termitophila* with a mass or progressive provisioning behavior, especially considering that some eumenines can be solitary and make small nests with a few cells but have, at the same time, progressive provisioning (e.g., some species of the genus *Synagris*).

#### 3.2.2 Proposed life cycle

Based on the evidence we collected from 23 *M. termitophila's* brood cells found inside 13 nests of *C. cyphergaster*, we hypothesize the life cycle of the new potter wasp species, shown in Figure 4. In this study, we did not observe all of the behaviors mentioned in the illustration. For mating, nest building and oviposition, specifically, we based our assumptions on the available literature described for the remainder species of Eumeninae (see “Potter wasps” in Section 2). Alternatively, *M. termitophila* may also nest in substrates other than a termite nest (e.g., in the ground, on top of rocks), as observed in other potter wasps. Yet, nesting inside termite nests and nesting on top of other structures are not mutually exclusive behaviors. Thus, a third hypothesis would be that the new wasp species could actually do both, having flexible nesting preferences.

## 4 DISCUSSION

In this report, for *M. termitophila*, we provided (a) the species description, (b) a proposed life cycle based on the documentation of active wasp brood cells within galleries of the termite nest, and (c) a first tentative evidence for a lack of conflict between adult wasps and termites. By itself, the wasp's strategy, characterized by the modification of termite nest's galleries into brood cells, constitutes a novel and uncommon form of nesting among potter wasps. While this behavior is seemingly a variant between the renter and digger types previously described (Iwata, 1976; Maindron, 1882), such a plasticity is in line with descriptions of other species belonging to Eumeninae (e.g., Cooper, 1979; Hermes et al., 2015; Krombein, 1979). To the best of our knowledge, this study is also the first to report wasp brood development occurring inside active termite colonies.

A fundamental question to be addressed about the emergence and stability of the association reported here is which underlying mechanisms allow *M. termitophila* wasps to develop offspring in the midst of active colonies of a termite, in this case, *C. cyphergaster*. We suspect that the exposition of wasps still in its early‐developing stages to the termite nest's substrate must have a major role in the evolution of such a nesting behavior. It has been generally hypothesized that once inside termite nests, termitophile species adopt chemical strategies to evade host detection, such as “chemical insignificance” (low concentration of recognition cues) or “chemical mimicry” (similarity in cuticular profiles) (Uboni et al., 2012). These mechanisms are known to impair interspecific detection in social insects (Rosa et al., 2018). With olfactory and tactile cues being arguably the basis of termite's intra‐ and interspecific recognition (Clement & Bagneres, 2019), colonies may eventually become more susceptible to nest invasion if members are unable to tell the difference between nest invaders and nestmates. Although more evidence is required to classify *M. termitophila* as a termitophile or termitariophile species, a chemical strategy for host deception would be possible regardless of the type of association between wasp and termites.

The knowledge about the biology of potter wasps is still incipient in many aspects, yet it seems straightforward to link the variety of nesting strategies observed within the group to unique selective pressures acting upon the different species. Accordingly, we suspect that *M. termitophila's* strategy for brood development is likely to be favored by a combination of two principal components: (i) plasticity in building behavior and (ii) flexibility in substrate preferences for nesting. Hence, the emergence of such a nesting behavior may be plausibly a variation derived from primitive forms, in which the wasp builds a similar structure elsewhere, and not on top of active termite colonies. It remains unclear whether such a development would be incidental or not, but in either case, the strategy may have been favored by selection, providing it confers adaptive value to the species. The wasp's nesting behavior may be also related to microclimate conditions or protection from predators and parasites, although with the current knowledge it is only possible to speculate in this regard. It seems worth mentioning that the way how *M. termitophila* uses *C. cyphergaster* nests somewhat resembles a host–commensal relationship, which goes in line with descriptions of other known nest intruders of this specific host termite species (Figure 1). Even though these species may be taxonomically independent, in most cases, they locate and use a suitable host nest to secure their offspring's development (for example, see Figure 1). A key point to be stressed here is that nest invaders known to complete their life cycle inside *C. cyphergaster* nests, seem to do so without impacting the colony, neither negatively nor positively.

Because this study is the first to record this specific wasp–termite association, drawing conclusions about associated costs and/or benefits is premature. That being said, we have reasons to suspect that *M. termitophila* does not negatively impact colonies of *C. cyphergaster*. For instance, we observed no evidence of conflict between both species when placing a recently ecloded wasp with a group of host termites (Video S1). Further inspection with more sampling will be necessary to confirm this observation and to test whether *M. termitophila* wasps never prey on termites. On the other hand, from a termite's perspective, it could be informative to develop a chemical analysis to investigate the both the larvae and the plastered mud we detected lining the internal walls of brood cells. Such a test would be particularly useful to confirm whether *M. termitophila* larvae have evolved chemicals to deter the termites and so avoid being detected inside the nest. In this same system (*C. cyphergaster*), host deterrence has been previously hypothesized based on the observation of inquiline termites (*I. microcerus*) that employ fecal pellets in response to the host termite's aggression (Hugo et al., 2020). We emphasize that none of the wasp brood cells we found (active or not) contained remnants of prey material. The absence of such remnants not only makes it difficult to fully confirm or deny *C. cyphergaster* termites as prey, but also prevents us from making further inferences about the wasp's preying strategies other than those already documented in the literature. Preying on termites would be a very unusual behavior for an eumenine wasp, since all currently known species hunt for caterpillars, beetles, and sometimes sawfly larvae. Although it remains open to investigation how exactly *M. termitophila* relates to the remainder described eumenines, we hypothesize that, as occurring in the other species (Krombein, 1979; Maindron, 1882), nectar must be a main source of nourishment for the adults. At the same time, it seems important to reiterate that we cannot rule out yet the possibility of termites as prey for nest provisioning. However, even in that case, with eumenine wasps exhibiting solitary habits and its larvae not demanding a great amount of prey to be fully nourished, it is unlikely that termite colonies would be negatively impacted to a substantial degree. If shown to be true, this set of traits should put *M. termitophila* in a position similar to previously described termitophiles, as a species that opportunistically benefit from a termite nest (e.g., being sheltered from predators) without causing substantial disruption or inflicting significant costs to the termite colony. Moreover, it remains to be further elucidated whether *M. termitophila* can nest in the ground or inside colonies of other termites, such as the remainder species belonging to Nasutitermitinae. In species of this subfamily, nests are not identical (Krishna et al., 2013; Noirot & Darlington, 2000) but still present architecture structurally comparable to *C. cyphergaster's*. Finally, further research focusing on understanding the mechanisms allowing the association between *M. termitophila* wasps and *C. cyphergaster* termites should provide insights into the selective pressures that have shaped the evolution of such an uncommon nesting behavior.

## CONFLICT OF INTEREST

None declared.

## AUTHOR CONTRIBUTION

**Helder Hugo:** Conceptualization (equal); Data curation (supporting); Funding acquisition (equal); Investigation (equal); Methodology (lead); Project administration (equal); Resources (equal); Visualization (equal); Writing‐original draft (equal); Writing‐review & editing (equal). **Marcel G. Hermes:** Conceptualization (equal); Data curation (equal); Investigation (equal); Methodology (equal); Resources (equal); Validation (equal); Visualization (equal); Writing‐original draft (equal); Writing‐review & editing (equal). **Bolívar R. Garcete‐Barrett:** Investigation (equal); Methodology (equal); Validation (equal); Writing‐review & editing (equal). **Iain D. Couzin:** Funding acquisition (equal); Resources (equal); Writing‐review & editing (equal).

## ETHICS STATEMENT

This study was conducted in compliance with relevant regulations governing animal research in Brazil, which includes (1) permits from the Brazilian Institute for the Environment and Renewable Natural Resources, (2) permission from landowners to conduct fieldwork on their property, and (3) tacit approval from the Brazilian Federal Government implied by employing authors to conduct scientific research. The termite species sampled is native from Brazil and currently is not listed as threatened or protected. No genetic information was accessed in this study.

## NOMENCLATURAL ACTS

The electronic edition of this article complies with the amended International Code of Zoological Nomenclature (ICZN), and therefore, the new name contained herein is available under that Code from the electronic edition of this article. This published work and the nomenclatural acts it contains have been registered in the online registration system for the ICZN, the ZooBank (LSID: zoobank.org:pub:58378E8D‐B5D3‐48A9‐9F53‐9B455717D875). The ZooBank LSIDs (Life Science Identifiers) can be resolved and the associated information viewed through any standard web browser by appending the LSID to the prefix “http://zoobank.org/”.

### Open Research Badges

This article has been awarded Open Materials, Open Data Badges. All materials and data are publicly accessible via the Open Science Framework at https://doi.org/10.5061/dryad.05qfttf1b.

## Supporting information

Figure S1

Figure S2

Table S1

Supplementary Material

APPENDIX

## Data Availability

We declare that all data supporting the conclusions of this study are available within the article and/or its Supplementary Information provided (Dryad, dataset, https://doi.org/10.5061/dryad.05qfttf1b). Nondigital data supporting the study (e.g., the holotype female used for species description, types used for species comparison) are deposited at Coleção Entomológica da Universidade Federal de Lavras, Minas Gerais, Brazil (CEUFLA), the Natural History Museum, London, England (NHM), and at the *Muséum National d’Histoire Naturelle*, Paris, France (MNHN). Upon reasonable request, further information related to this study can be requested from the corresponding author.
