# Corrigendum

**DOI:** 10.1002/ece3.7859

**Published:** 2021-07-22

In the recent article by Hugo et al. (2020), the word “ecloded” was incorrectly used. The correct term should have been “eclosed.” The error occurs three times throughout the text: in the Abstract, Section 2.5 in Material and Methods, and paragraph 4 of the Discussion. Also, in Section 2.4, the word “ecloding” should be changed to “eclosing”. The details are as follows:

### Abstract

The sentence “Besides scientifically describing *Montezumia termitophila* sp. nov. (Vespidae: Eumeninae), named after its association with the termite *Constrictotermes cyphergaster* (Silvestri, 1901) (Termitidae: Nasutitermitinae), we provide preliminary information about the new species' bionomics by including (a) a hypothetical life cycle based on the evidence we collected and (b) a footage showing the first interaction between a recently ecloded wasp and a group of termites” should read “Besides scientifically describing *Montezumia termitophila* sp. nov. (Vespidae: Eumeninae), named after its association with the termite *Constrictotermes cyphergaster* (Silvestri, 1901) (Termitidae: Nasutitermitinae), we provide preliminary information about the new species' bionomics by including (a) a hypothetical life cycle based on the evidence we collected and (b) a footage showing the first interaction between a recently ecloded wasp and a group of termites.”

## 2.4 | Study site, sampling, and nest inspection

The sentence “As for verifying whether *M*. *termitophila's* nesting takes place entirely inside *C*. *cyphergaster* nests, it would be necessary to (a) obtain empirical evidence of adult wasps ecloding from the inside of termite nests and (b) document the presence of wasp's offspring in termite nests, by detecting wasp brood cells integrated between termite galleries containing wasp immature stages” should read “As for verifying whether *M*. *termitophila's* nesting takes place entirely inside *C*. *cyphergaster* nests, it would be necessary to (a) obtain empirical evidence of adult wasps eclosing from the inside of termite nests and (b) document the presence of wasp's offspring in termite nests, by detecting wasp brood cells integrated between termite galleries containing wasp immature stages.”

## 2.5 | Recording wasp–termite interaction

The sentence “As such, it provides a first tentative empirical evidence for a mutual lack of aggression between a recently ecloded *M*. *termitophila* wasp and its host termites” should read “As such, it provides a first tentative empirical evidence for a mutual lack of aggression between a recently eclosed *M*. *termitophila* wasp and its host termites.”

## 4 | DISCUSSION

In paragraph 4 of the Discussion, the sentence “For instance, we observed no evidence of conflict between both species when placing a recently ecloded wasp with a group of host termites (Video S1)” should read “For instance, we observed no evidence of conflict between both species when placing a recently eclosed wasp with a group of host termites (Video S1).”
