# Supplementary figures and images for: First evidence of wasp brood development inside active nests of a termite with the description of a previously unknown potter wasp species
Source: Ecol Evol. 2020 Oct 6;10(23):12663–74. doi: 10.1002/ece3.6872 (PMC7713954; doi:10.1002/ece3.6872)

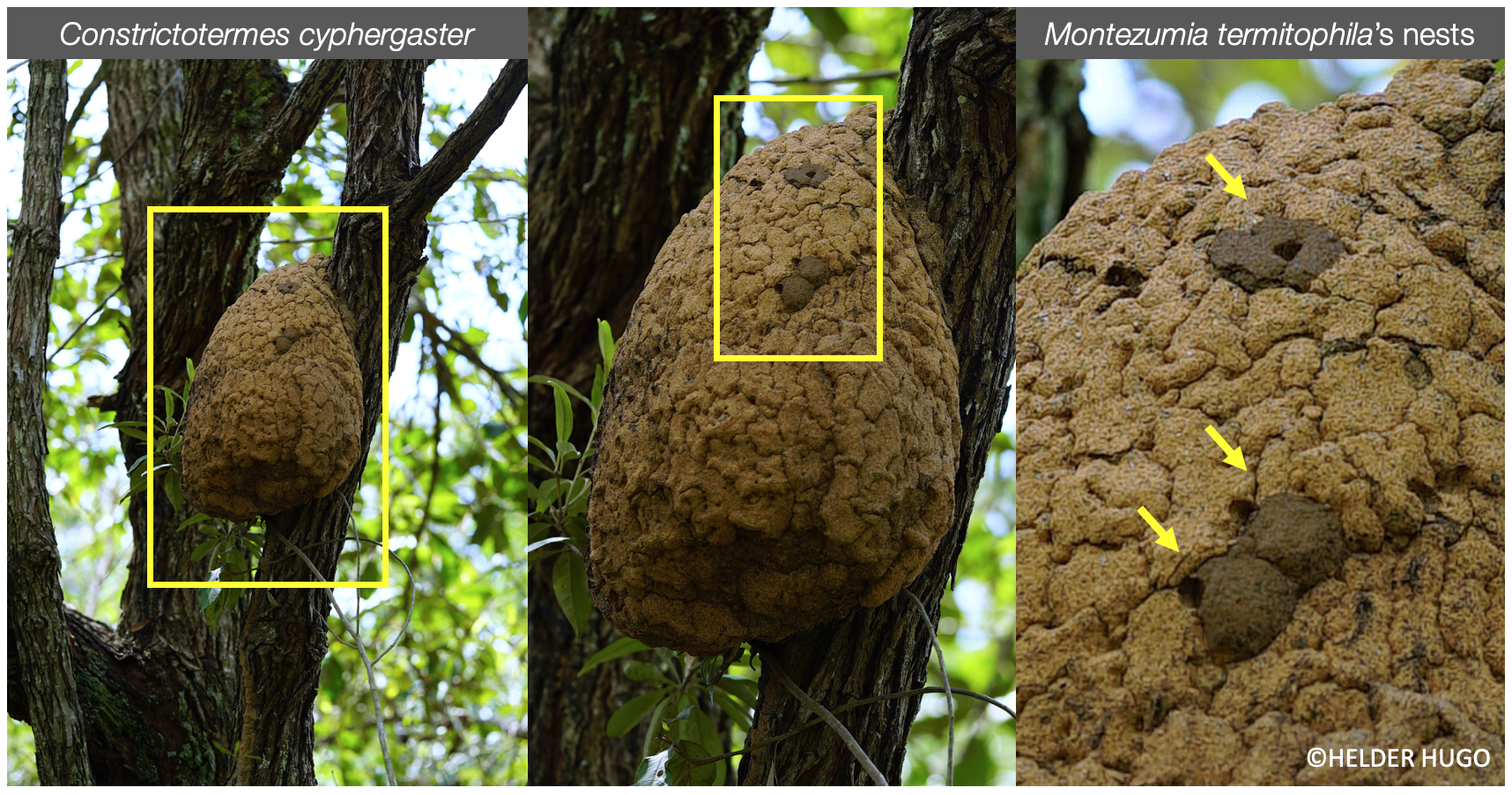

Supplement: Supplementary file 1 — Figure S1 [file ECE3-10-12663-s001.tif]

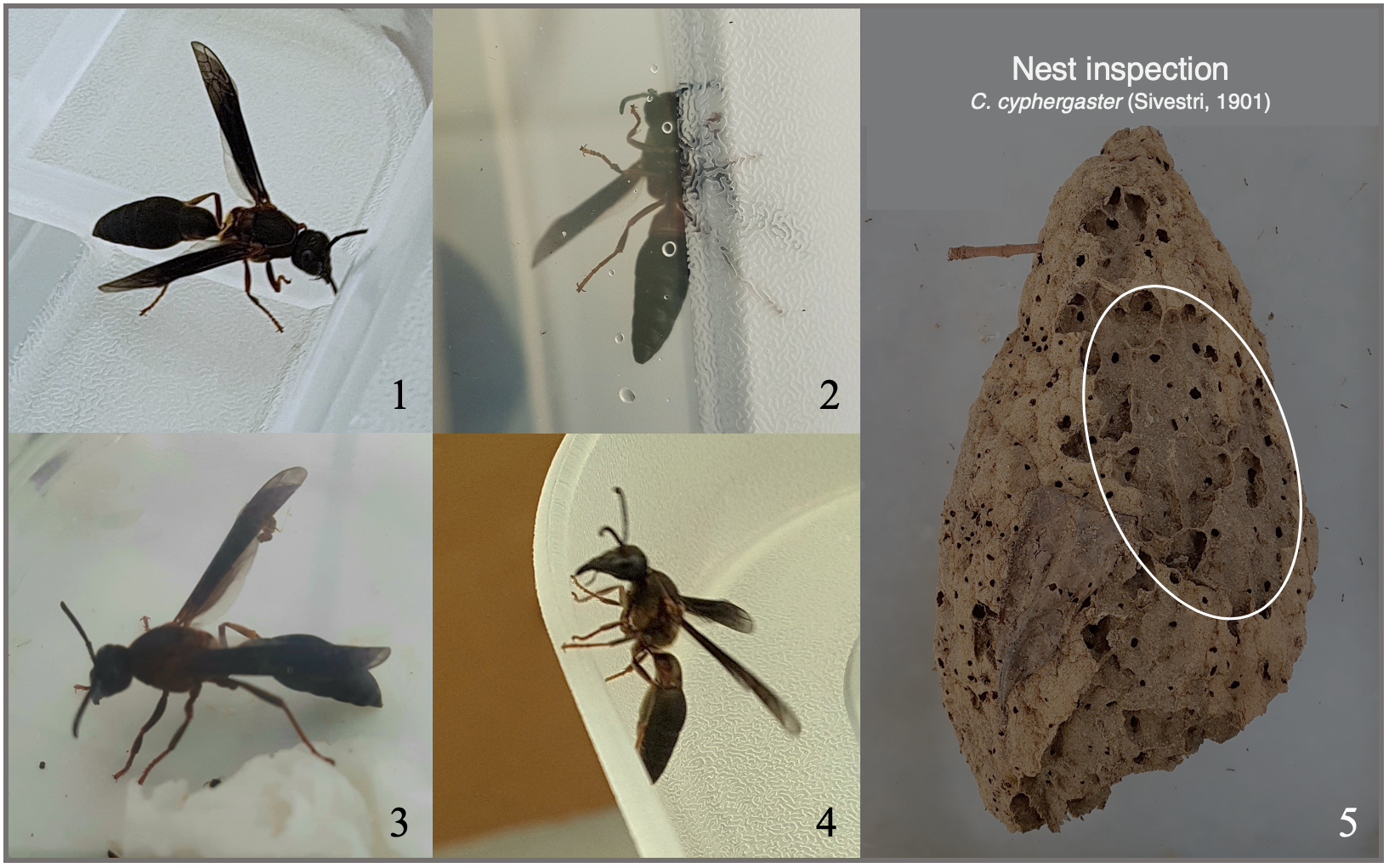

Supplement: Supplementary file 2 — Figure S2 [file ECE3-10-12663-s002.tif]
