# Supplementary material for: First evidence of wasp brood development inside active nests of a termite with the description of a previously unknown potter wasp species
Source: Ecol Evol. 2020 Oct 6;10(23):12663–74. doi: 10.1002/ece3.6872 (PMC7713954; doi:10.1002/ece3.6872)
Supplement: Supplementary file 3 — Table S1 [file ECE3-10-12663-s003.docx]

|  | **Fieldwork - Constrictotermes cyphergaster (Silvestri 1901) - Divinopolis-MG, Brazil, Mar-Apr 2018 – Helder Hugo** | | | | | | | | | | | | | | | | | | | | | |
| --- | --- | --- | --- | --- | --- | --- | --- | --- | --- | --- | --- | --- | --- | --- | --- | --- | --- | --- | --- | --- | --- | --- |
|  | **NestID** | **H** | **T** | **M** | **B** | **r (M/2πR)** | **h (H/4)** | **SphericalCap** | **Cilinder** | **NestVolume** | **Litre** | **NestBuilder** | **CollectionSite** | **CollectionDate** | **CollectedBy** | **InactiveBroodCells** | **ActiveBroodCells** | **LarvalStage** | **Morphotype** | **SpecifesIdentification** | **IdentifiedBy** | **TypeSpecimen** |
| **1** | N01HHS2018 | *40* | *73* | *89* | *83* | *14.17* | *10* | *3403.33* | *12613.06* | *19419.72* | *19.42* | *C. cyphergaster* | Divinópolis-MG, Brazil | 25.3.2018 | HH | 0 | 0 |  |  |  |  |  |
| **2** | N02HHS2018 | *18* | *36* | *43* | *36* | *6.85* | *4.5* | *340.00* | *1324.92* | *2004.92* | *2.00* | *C. cyphergaster* | Divinópolis-MG, Brazil | 25.3.2018 | HH | 2 | 0 |  |  |  |  |  |
| **3** | N03HHS2018 | *33* | *53* | *65* | *58* | *10.35* | *8.25* | *1624.31* | *5550.36* | *8798.98* | *8.80* | *C. cyphergaster* | Divinópolis-MG, Brazil | 25.3.2018 | HH | 1 | 1 | larvae | Eumeninae-01 | *M. termitophila* sp nov. | MGH & BRGB | (Female holotype) |
| **4** | N04HHS2018 | *30* | *55* | *70* | *55* | *11.15* | *7.5* | *1527.19* | *5851.91* | *8906.29* | *8.91* | *C. cyphergaster* | Divinópolis-MG, Brazil | 25.3.2018 | HH | 3 | 0 |  |  |  |  |  |
| **5** | N05HHS2018 | *32* | *36* | *50* | *48* | *7.96* | *8* | *1064.11* | *3184.71* | *5312.93* | *5.31* | *C. cyphergaster* | Divinópolis-MG, Brazil | 25.3.2018 | HH | 3 | 0 |  |  |  |  |  |
| **6** | N06HHS2018 | *33* | *49* | *69* | *65* | *10.99* | *8.25* | *1760.44* | *6254.50* | *9775.37* | *9.78* | *C. cyphergaster* | Divinópolis-MG, Brazil | 25.3.2018 | HH | 1 | 0 |  |  |  |  |  |
| **7** | N07HHS2018 | *38* | *33* | *60* | *68* | *9.55* | *9.5* | *1810.11* | *5445.86* | *9066.09* | *9.07* | *C. cyphergaster* | Divinópolis-MG, Brazil | 27.3.2018 | HH & JHS | 1 | 0 |  |  |  |  |  |
| **8** | N08HHS2018 | *30* | *53* | *66* | *70* | *10.51* | *7.5* | *1414.69* | *5202.23* | *8031.60* | *8.03* | *C. cyphergaster* | Divinópolis-MG, Brazil | 27.3.2018 | HH & JHS | 0 | 1 | pupae | Eumeninae-01 | *M. termitophila* sp nov. |  |  |
| **9** | N09HHS2018 | *35* | *67* | *76* | *66* | *12.10* | *8.75* | *2208.19* | *8047.77* | *12464.15* | *12.46* | *C. cyphergaster* | Divinópolis-MG, Brazil | 27.3.2018 | HH & JHS | 1 | 0 |  |  |  |  |  |
| **10** | N10HHS2018 | *34* | *69* | *87* | *77* | *13.85* | *8.5* | *2500.09* | *10244.67* | *15244.85* | *15.24* | *C. cyphergaster* | Divinópolis-MG, Brazil | 27.3.2018 | HH & JHS | 2 | 1 | pupae | Eumeninae-01 | *M. termitophila* sp nov. |  |  |
| **11** | N11HHS2018 | *34* | *60* | *67* | *70* | *10.67* | *8.5* | *1777.59* | *6075.88* | *9631.06* | *9.63* | *C. cyphergaster* | Divinópolis-MG, Brazil | 27.3.2018 | HH & JHS | 1 | 0 |  |  |  |  |  |
| **12** | N12HHS2018 | *39* | *71* | *93* | *96* | *14.81* | *9.75* | *3450.29* | *13427.99* | *20328.57* | *20.33* | *C. cyphergaster* | Divinópolis-MG, Brazil | 27.3.2018 | HH & JHS | 0 | 0 |  |  |  |  |  |
| **13** | N13HHS2019 | *45* | *75* | *89* | *88* | *14.17* | *11.25* | *4141.76* | *14189.69* | *22473.21* | *22.47* | *C. cyphergaster* | Divinópolis-MG, Brazil | 27.3.2018 | HH & JHS | 4 | 1 | pupae | Eumeninae-01 | *M. termitophila* sp nov. |  |  |
