# Supplementary material for: First evidence of wasp brood development inside active nests of a termite with the description of a previously unknown potter wasp species
Source: Ecol Evol. 2020 Oct 6;10(23):12663–74. doi: 10.1002/ece3.6872 (PMC7713954; doi:10.1002/ece3.6872)
Supplement: Supplementary file 5 — APPENDIX [file ECE3-10-12663-s005.pdf]

APPENDIX - Supporting information

First evidence of wasp brood development inside active nests of a termite with the description of a previously unknown potter wasp species

Hugo, H, Hermes, MG, Garcete-Barrett, BR, Couzin, ID. First evidence of wasp brood development inside active nests of a termite with the description of a previously unknown potter wasp species. *Ecol Evol* 2020; 00: 1– 12. https://doi.org/10.1002/ece3.6872

First published: 06 October 2020

Video S1. Interaction between female *Montezumia termitophila* and workers and soldiers of *Constrictotermes cyphergaster*; observation Available in DRYAD: https://doi.org/10.5061/dryad.05qfttf1b

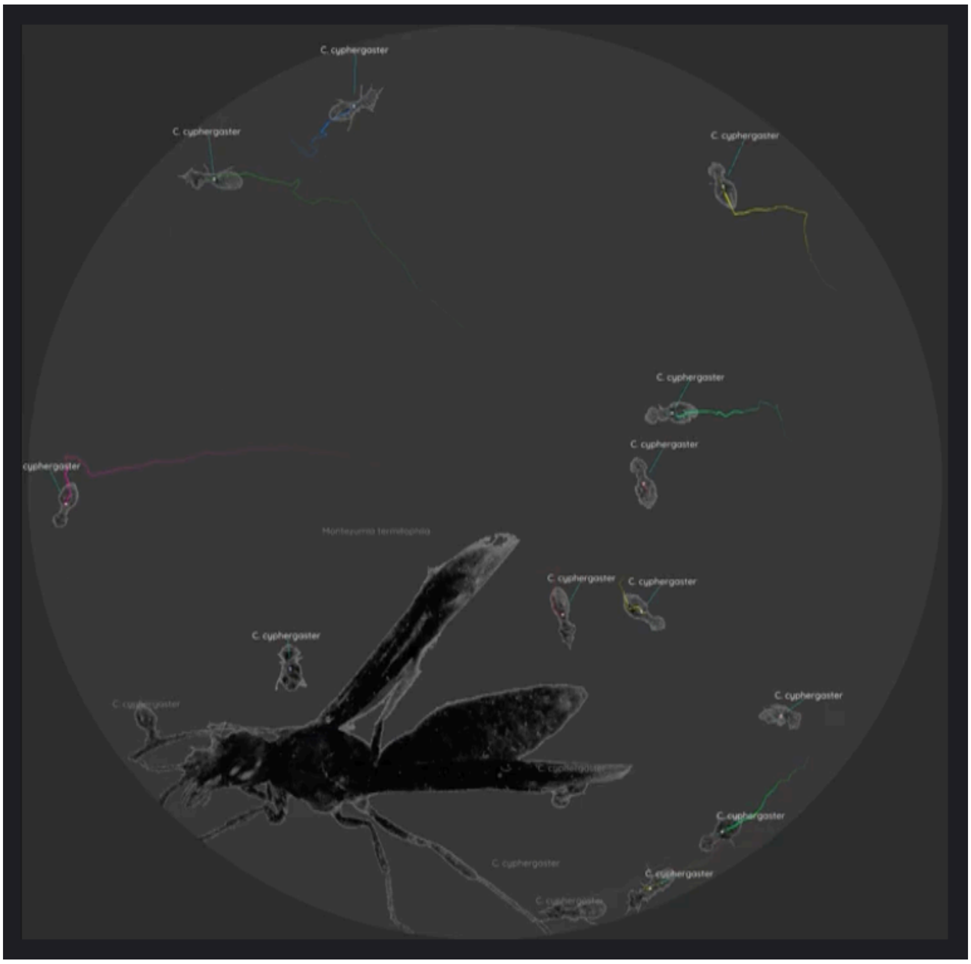

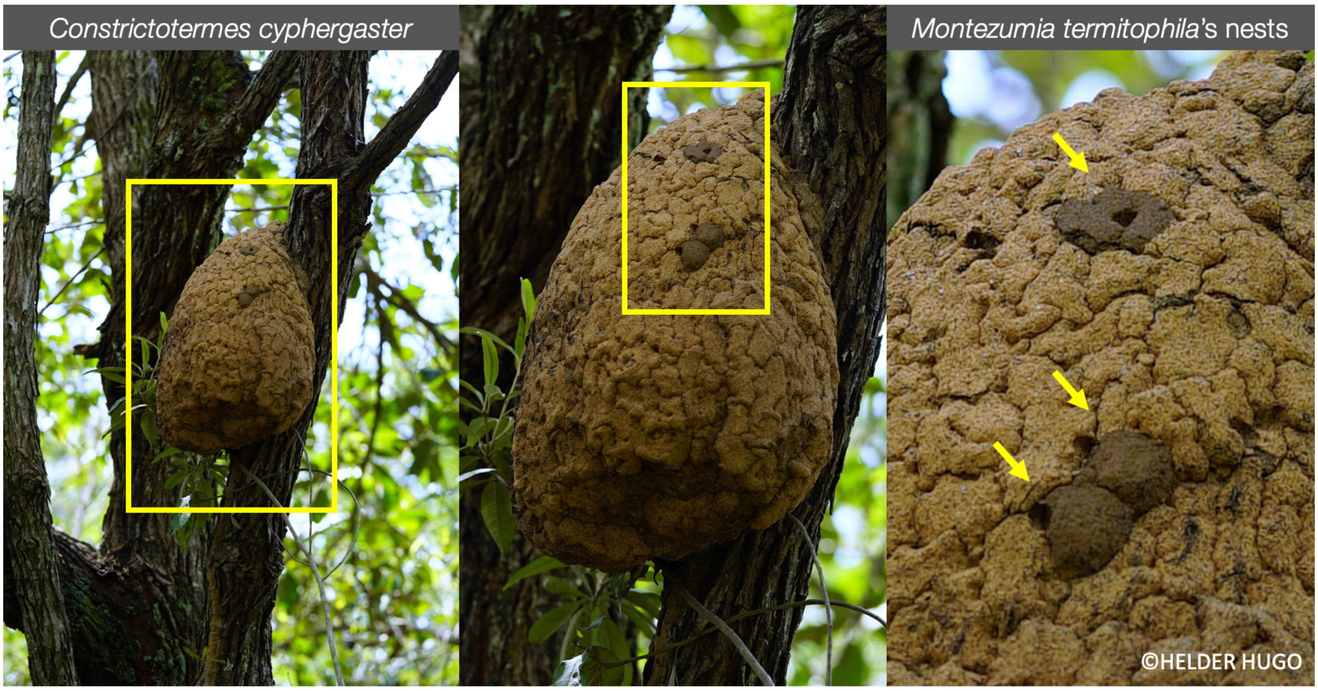

Figure S1. Representative example of arboreal nests of *C. cyphergaster*. Markings in yellow highlights three *M. termitophila*'s brood cells present on the nest surface.

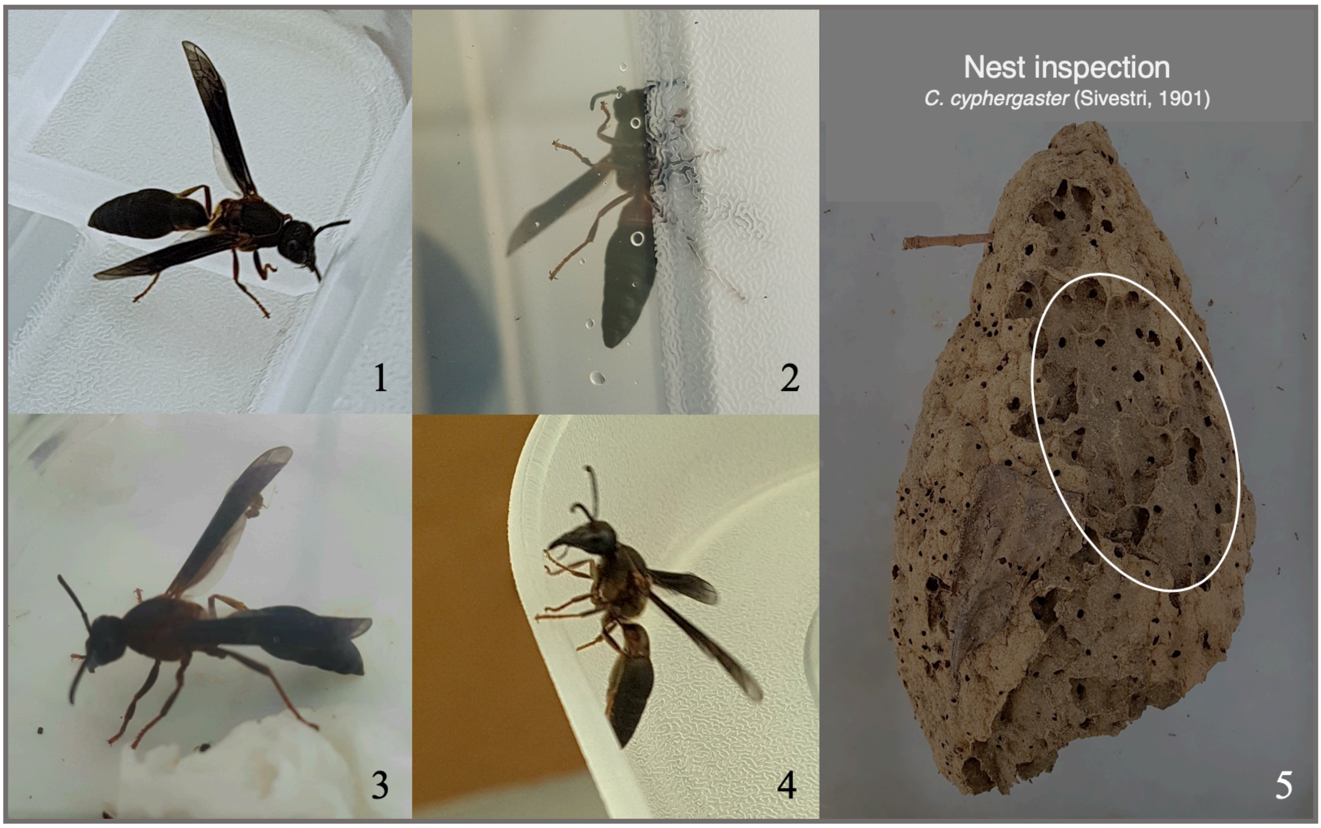

Figure S2. Adults of *M. termitophila* emerged from brood cells found in *C. cyphergaster* nests. (1) Female; (2) Sex not confirmed; (3) Female holotype; (4) Male; (5) Example of termite nest having its galleries exposed layer by layer during nest inspection. The section already exposed using scoopulas is highlighted.
